# *Pneumocystis* pneumonia in HIV-positive patients in Spain: epidemiology and environmental risk factors

**DOI:** 10.7448/IAS.18.1.19906

**Published:** 2015-05-20

**Authors:** Alejandro Alvaro-Meca, Ines Palomares-Sancho, Asuncion Diaz, Rosa Resino, Angel Gil De Miguel, Salvador Resino

**Affiliations:** 1 Department of Preventive Medicine & Public Health, Rey Juan Carlos University, Madrid, Spain; 2 Unit of HIV Surveillance and Behavioural Monitoring, National Centre of Epidemiology, Institute of Health Carlos III, Madrid, Spain; 3 Network of Biomedical Research Centres Epidemiology and Public Health (Centro de Investigación Biomédica en Red de Epidemiología y Salud Pública (CIBERESP), Madrid, Spain; 4 Department of Human Geography, Faculty of Geography and History, Complutense University of Madrid, Madrid, Spain; 5 Unit of Viral Infection and Immunity, National Centre for Microbiology, Institute of Health Carlos III, Majadahonda, Madrid, Spain

**Keywords:** *Pneumocystis*, epidemiology, seasonality, air pollution, AIDS

## Abstract

**Introduction:**

Specific environmental factors may play a role in the development of *Pneumocystis* pneumonia (PCP) in HIV-positive patients. The aim of this study was to estimate the PCP incidence and mortality in hospitalized HIV-positive patients in Spain during the combination antiretroviral therapy (cART) era (1997 to 2011), as well as to analyze the climatological factors and air pollution levels in relation to hospital admissions and deaths.

**Methods:**

We carried out a retrospective study. Data were collected from the National Hospital Discharge Database and the State Meteorological Agency of Spain. A case-crossover analysis was applied to identify environmental risk factors related to hospitalizations and deaths. For each patient, climatic factors and pollution levels were assigned based on readings from the nearest meteorological station to his or her postal code.

**Results:**

There were 13,139 new PCP diagnoses and 1754 deaths in hospitalized HIV-positive patients from 1997 to 2011. The PCP incidence (events per 1000 person-years) dropped from 11.6 in 1997 to 2000, to 5.4 in 2004 to 2011 (*p*<0.001). The mortality (events per 10,000 person-years) also decreased from 14.3 in 1997 to 2000, to 7.5 in 2004 to 2011 (*p*<0.001). Most hospital admissions and deaths occurred in the winter season and the fewest occurred in the summer, overlapping respectively with the lowest and highest temperatures of the year in Spain. Moreover, lower temperatures prior to PCP admission, as well as higher concentrations of NO_2_ and particulate matter up to 10 m in size (PM10) at the time of admission were associated with higher likelihoods of hospital admission due to PCP when two weeks, one month, 1.5 months or two months were used as controls (*p*<0.01). Furthermore, higher concentrations of ozone at one month (*p=*0.007), 1.5 months (*p*<0.001) and two months (*p=*0.006) prior to admission were associated with higher likelihoods of hospital admission with PCP. For PCP-related deaths, lower temperatures prior to admission and higher concentrations of atmospheric PM10 at the time of admission were related to higher likelihood of death when two weeks, one month and 1.5 months were used as controls (*p*<0.05).

**Conclusions:**

PCP was a significant health problem in the cART era (1997 to 2011), and PCP epidemiology was adversely influenced by colder climatological factors and higher ambient air pollution levels.

## Introduction

The fungus *Pneumocystis jirovecii* causes *Pneumocystis* pneumonia (PCP), one of the most frequent and serious opportunistic infections in HIV-positive individuals, and is a leading cause of death worldwide [1]. In the European Union, PCP was the most common AIDS-indicative disease diagnosed in 2012 [2], but its incidence has decreased since the introduction of chemoprophylaxis and combination antiretroviral therapy (cART) [3,4]. PCP chemoprophylaxis is recommended for HIV-positive patients if their CD4+ cell count is below 200 cells/mm^3^ or if they have a history of oral candidiasis (primary prophylaxis), and after an episode of PCP (secondary prophylaxis) [5]. However, PCP remains a major health concern for individuals unaware that they are HIV positive or those that do not have access to cART, are unable to tolerate the treatment, or for whom cART is not effective [1,2,6].

*P. jirovecii* is ubiquitous and data show worldwide seroprevalence in individuals starting from an early age [7]. Therefore, *P. jirovecii* likely colonizes the host lung for extended periods of time with a low microorganism burden and without signs or symptoms of PCP. This most likely precedes the development of acute PCP [7]. The duration of this colonization and the events that trigger PCP requiring hospitalization are poorly understood [7]. The most important factors related to the occurrence of PCP may be classified into three groups: 1) host factors (e.g. CD4+ T cell counts [8]); 2) microorganism factors (e.g. *Pneumocystis* genotypes and virulence factors [9]); and 3) environmental factors (e.g. climatological factors and air pollutants [10,11]).

The role that specific environmental factors may play in the development of PCP in HIV-positive patients has piqued scientific interest in recent years [11–13], because climatological factors and air pollution levels have been shown to have an impact on the progression of several pulmonary diseases [14]. However, published data on environmental factors in relation to PCP disease are somewhat inconsistent. Conclusions about the seasonal effects on PCP incidence have been variable across several studies, with peaks of PCP incidence in both summer and winter months or no seasonal variation at all [11,15–18]. Moreover, the effects of air pollution on PCP are still poorly understood. In a recent paper, Djawe *et al*. [11] analyzed the effects of increased levels of air pollutants in a cohort of HIV-positive patients from San Francisco, finding that sulfur dioxide (SO_2_) was related to higher incidence of hospital admissions for PCP, whereas no relationship was found for carbon monoxide (CO), nitrogen dioxide (NO_2_), ozone (O_3_) or particulate matter up to 10 µm in size (PM10). In addition, Blount *et al*. [13] reported that elevated atmospheric concentrations of PM10 and NO_2_ were associated with a suppressed IgM response to a recombinant *Pneumocystis* major surface glycoprotein, which may cause longer hospital stays.

The aim of this study was to estimate PCP incidence and mortality in hospitalized HIV-positive patients with a PCP diagnosis in Spain between 1997 and 2011 and to analyze climatological factors and air pollution levels in relation to hospital admissions and deaths.

## Materials and methods

### Study population

We carried out a retrospective study of all HIV-positive patients over 15 years of age with a discharge diagnosis of PCP disease in Spain from 1 January 1997 to 31 December 2011. Data were obtained from the records of the Minimum Basic Data Set (MBDS) provided by the Spanish Ministry of Health.

The MBDS is a clinical and administrative database containing information obtained at discharge, with an estimated coverage of 97.7 and 25% of total hospital admissions to public and private hospitals, respectively [19]. However, all HIV-positive patients are treated through the public health system in Spain and thus are likely to receive their care at public hospitals.

The MBDS provided the encrypted patient identification number, sex, date of birth, dates of hospital admission and discharge, patients’ residential postal code, medical institutions providing the services, the International Classification of Diseases, 9th ed., Clinical Modification (ICD-9-CM) codes of diagnoses and procedures, and outcome at discharge. A hospitalization was defined as a discharge record in the MBDS.

The data were treated with full confidentiality, according to Spanish legislation. Patient identifiers were deleted before the database was provided to the authors in order to keep strict patient confidentiality. Given the anonymous and mandatory nature of the data, informed consent was neither required nor necessary. The Spanish Ministry of Health evaluated the protocol of our investigation and considered that it met all ethical aspects according to Spanish legislation. The study was approved by the Research Ethic Committee of the Instituto de Salud Carlos III (Madrid, Spain).

### Environmental data

We did not have data to identify individual exposure measurements, but exposure to climatic and pollutant factors were obtained indirectly via the postal codes of all patients included at the time of hospitalization. The date of hospital admission and death were divided into four seasons: spring (March to May), summer (June to August), autumn (September to November) and winter (December to February).

Environmental data were obtained by the State Meteorological Agency (AEMET) of Spain, (www.aemet.es/) provided by the Spanish Ministry of Agriculture, Food and Environment. For each station, AEMET provided daily data from 1 January 1996 to 31 December 2011 (temperature, humidity, SO_2_, NO_2_, O_3_, PM10, CO and station geolocation (latitude, longitude and altitude)). For each patient, climatic and pollutant factors were assigned using the nearest meteorological station to his or her postal code.

### Calendar periods

cART is standard treatment for HIV-positive patients and the number of patients receiving cART has been steadily increasing since 1996 [20–22]. In this study, we divided the follow-up period from 1997 to 2011 into three periods, according to the widespread use of cART:

1. Early-period cART from 1997 to 1999, in which patients received the first protease inhibitors (PIs) introduced (i.e. indinavir, saquinavir, ritonavir, saquinavir, etc.). These cART regimens had low adherence and a high number of side effects.
2. Mid-period cART from 2000 to 2003, in which there was a simplification of cART regimens (i.e. liponavir/ritonavir, efavirenz, etc.), which permitted twice-daily medication dosing, lower overall pill burden and fewer food restrictions.
3. Late-period cART from 2004 to 2011, in which new cART regimens based on coformulation of antiretroviral drugs were initiated (i.e. Atripla, Truvada, Kivexa, etc.) permitting even lower pill burden, once daily dosing and fewer food restrictions, which resulted in increased adherence and effectiveness of cART.

### Outcome variables

The index episode was defined as the occurrence of a hospital discharge with PCP diagnosis according to the ICD-9-CM (code: 136.3). The outcome variables analyzed in this study were: 1) hospital admissions with a new PCP diagnosis (incident case); and 2) death with a PCP diagnosis.

Hospital readmissions during the first 30 days since the first admission were not counted as new episodes of PCP. Incidences of patients who were readmitted with a PCP diagnosis 30 days after their first admission were counted as a new episode of PCP disease.

### Statistical analysis

A retrospective design was used to evaluate the trend in PCP incidence and mortality. For the calculation of rates, the number of events within each calendar year was used as the numerator, and the denominator was the estimate of the number of people living with HIV/AIDS in Spain provided by the National Centre of Epidemiology (Instituto de Salud Carlos III, Madrid, Spain) [23]. The incidence and mortality were calculated according to the three calendar periods described above.

A case-crossover design (CCD) was used to evaluate the effect of each environmental factor on the two clinical outcomes analyzed in this study (new PCP diagnosis and death with a PCP diagnosis). In the CCD, each individual experiencing a health event serves as his own control [24,25]. In air pollution epidemiology, CCD is the most suitable study design for studying the effects on health outcomes of varying short-term exposure [26], and it has been used in a large number of epidemiological studies about environmental factors and respiratory diseases [11,27,28]. In the case of PCP, the period from the first appearance of PCP symptoms to the time of hospital admission is about four to eight weeks in HIV-positive patients [29]. In our study, four different time points before hospital admission (two weeks, one month, one-and-a-half months, and two months) were selected to compare the environmental exposure of individual patients to the time of presentation with PCP diagnosis (baseline or week 0). For each time point, and following the example set by Djawe *et al*. [11], we looked at an average value for each environmental factor over a three-day period (on the day of PCP diagnosis and the two days immediately prior) in order to eliminate an “extreme” level. Finally, conditional logistic regression (CLR) was used to evaluate the association between environmental factors and clinical outcomes. The odds ratio (OR) and its 95% confidence interval were calculated by exact method. In a CCD for each factor, the odds of event with respect to an increase in the average level of the environmental factor around the date of hospitalization (case at hospital admission) were compared to the change in the factor when PCP hospitalization did not occur (control time at two weeks, one month,1.5 months and two months before hospital admission). In the basic inference, for each case the exposure status during the admission (encoded as “1”) and control time (encoded as “0”) are compared, and only subjects with different levels of exposure are informative [26]. In our study, OR values higher than one indicate the analyzed factor is associated with higher risk when it is increased at the time of hospital admission or decreased before hospital admission, whereas OR values lower than one indicate the analyzed factor is associated with greater risk when it is increased at the control time (two weeks, one month, 1.5 months and two months before hospital admission) or decreased at the time of hospital admission. All environmental factors, except temperature, were log-transformed because they varied greatly across dates of measurements.

The seasonal effect on hospital admissions and death related to PCP was evaluated using a Bayesian temporal model with Poisson distribution [30]. Significance of seasonal effect was calculated based on deviance information criterion [31].

Statistical analysis was performed using the R statistical package (version 3.1.0) [32]. All tests were two-tailed with *p* <0.05 considered significant.

## Results

### Characteristics of study population

Table 1 shows the clinical and epidemiological characteristics of patients included in the study period. At admission, their mean age was 39.03 years. A large majority were men (75.9%) with a mean hospital stay of 20.88 days and mean Charlson comorbidity index of 0.48. The most common comorbidities were mild liver disease (22.4%), chronic pulmonary disease (5.7%) and cancer (4.1%) (Table 1).

**Table 1:** Summary of epidemiological and clinical characteristics of HIV-positive patients with a *Pneumocystis* pneumonia diagnosis from 1997 to 2011 in Spain

Description	Data
No. of patients	13,139
Males^a^	9978 (75.9%)
Age (years)^b^	39.0 (9.2)
Length of stay (days)^b^	20.9 (18.6)
Charlson index^b^	0.5 (0.8)
Conditions influencing health status^a^	
Surgical conditions	131 (1%)
Trauma	76 (0.6%)
Comorbid diseases^a^	
Myocardial infarction	38 (0.3%)
Congestive heart failure	114 (0.9%)
Peripheral vascular disease	37 (0.3%)
Cerebrovascular disease	94 (0.7%)
Dementia	100 (0.8%)
Chronic pulmonary disease	752 (5.7%)
Connective tissue disease-rheumatic disease	10 (0.1%)
Peptic ulcer disease	53 (0.4%)
Mild liver disease	2944 (22.4%)
Moderate or severe liver disease	135 (1%)
Paraplegia and hemiplegia	85 (0.6%)
Renal disease	123 (0.9%)
Cancer	537 (4.1%)
Metastatic carcinoma	35 (0.3%)

a Absolute number (percentage)

b mean (standard deviation).

### Epidemiology of PCP among HIV-positive patients in Spain

**Figure 1:**
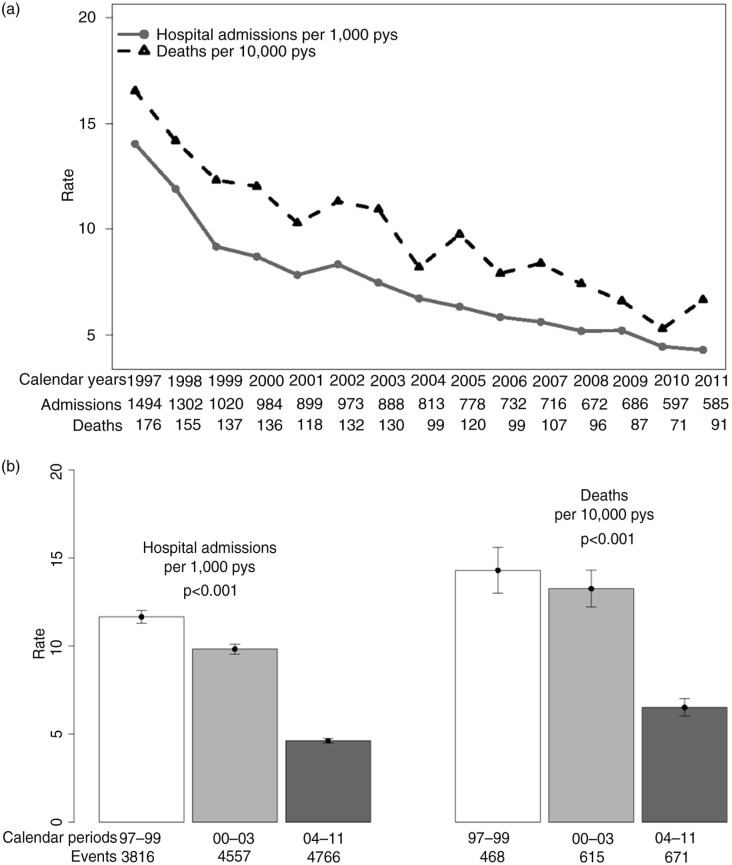
Trends of PCP diagnosis rate and death in Spain (1997 to 2011) according to calendar year (a) and cART period (b). PCP: *Pneumocystis* pneumonia.

There were 13,139 new PCP diagnoses from 1997 to 2011; the overall rate of PCP in all HIV- positive patients (events per 1000 persons/year) was 7.2 (95% confidence interval (95%CI)=7.1; 7.3). When we divided the follow-up period by calendar years, hospital admission rates of PCP showed a significant decrease from 1997 to 2011 (Figure 1a *p*<0.001). For analysis by cART period (Figure 1b), PCP incidence decreased from 11.6 (95%CI=11.3; 12.0) in 1997 to 2000, to 8.1 (95%CI=7.8; 8.3) in 2000 to 2003 (*p<*0.001), and then to 5.4 (95%CI=5.3; 5.6) in 2004 to 2011 (*p*<0.001).

There were 1754 deaths in HIV-positive patients with PCP from 1997 to 2011. The overall rate of mortality in all HIV-positive patients with PCP (events per 10,000 persons/year) was 9.6 (95%CI=9.2; 10.1). By calendar year, the mortality rate showed a significant decrease from 1997 to 2011 (Figure 1a; *p*<0.001). When PCP was analyzed across cART periods (Figure 1b), mortality also had a dramatic decrease from 14.3 (95%CI=13.0; 15.6) in 1997 to 2000, to 11.1 (95%CI=10.2; 12.1) in 2000 to 2003 (*p*<0.001), and finally to 7.5 (95%CI=6.9; 8.0) in 2004 to 2011 (*p*<0.001).

### Seasonality of hospital admissions and deaths related to PCP

**Figure 2:**
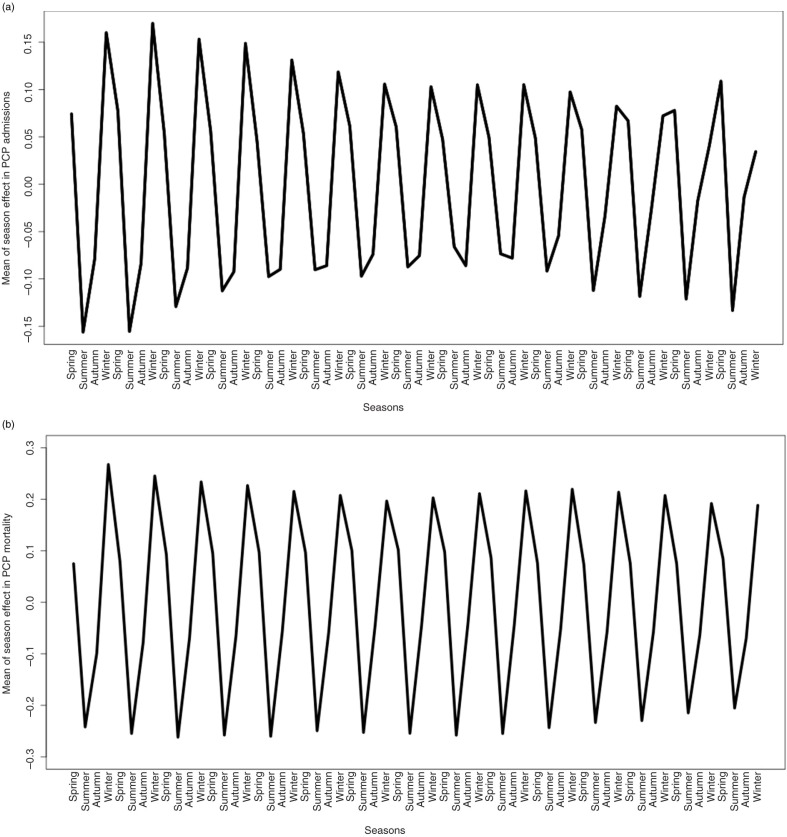
(a) Mean of the seasonal effect of PCP diagnosis rate in Spain between 1997 and 2011. (b) Mean of the seasonal effect of PCP mortality rate in Spain between 1997 and 2011. PCP, *Pneumocystis* pneumonia.

A significant seasonal effect was found during the period of study on hospital admissions (Figure 2a) and deaths (Figure 2b). The lowest values of hospital admissions and deaths were found in summer and most of the hospital admissions and deaths occurred in winter, which coincided with the lowest and highest temperatures of the year in Spain, respectively (Figure 2a and b). Moreover, the amplitude of the seasonal effect slightly declined over time for hospital admissions (Figure 2a), whereas it remained constant for deaths (Figure 2b).

### Effect of environmental factors on PCP admission and death

Table 2 shows the relationship between each environmental factor and PCP hospital admissions. In a multivariate CLR analysis, lower temperatures prior to PCP admission, as well as higher concentrations of NO_2_ and PM10 at the time of admission were significantly associated with higher likelihoods of PCP hospital admissions when two weeks, one month, 1.5 months or two months were used as controls (*p*<0.01) (Table 2). Furthermore, higher concentrations of ozone at one month (*p*=0.007), 1.5 months (*p*<0.001) and two months (*p*=0.006) prior to PCP admission, and CO at two months (*p*<0.001), were significantly associated with higher likelihoods of PCP hospital admissions (Table 2). The effects of humidity and SO_2_ were not statistically significant.

**Table 2:** Summary of the association between environmental factors and hospital admissions for PCP in HIV-positive patients using two weeks, one month, 1.5 months and two months before hospital admission as controls

	Univariate		Multivariate	
		
Environmental exposure (units)	OR (95% CI)	*p*	OR (95% CI)	*p*
Two weeks				
Temperature (°C)	1.03 (1.01; 1.05)	**<0.001**	1.03 (1.01; 1.05)	**<0.001**
Humidity (×10%)	1.00 (0.98; 1.02)	0.994	0.99 (0.97; 1.01)	0.559
NO_2_ (µg/m^3^)	1.19 (1.13; 1.26)	**<0.001**	1.17 (1.10; 1.25)	**<0.001**
SO_2_ (µg/m^3^)	1.05 (1.01; 1.10)	**0.017**	1.00 (0.96; 1.05)	0.882
O_3_ (µg/m^3^)	0.94 (0.88; 0.99)	**0.028**	0.96 (0.90; 1.03)	0.250
PM10 (µg/m^3^)	1.11 (1.06; 1.16)	**<0.001**	1.07 (1.02; 1.12)	**0.004**
CO (µg/m^3^)	1.04 (0.99; 1.10)	0.113	0.97 (0.92; 1.03)	0.317
One month				
Temperature (°C)	1.02 (1.01; 1.03)	**<0.001**	1.02 (1.01; 1.03)	**<0.001**
Humidity (×10%)	0.99 (0.97; 1.01)	0.249	0.98 (0.96; 1.00)	0.067
NO_2_ (µg/m^3^)	1.18 (1.12; 1.25)	**<0.001**	1.18 (1.11; 1.25)	**<0.001**
SO_2_ (µg/m^3^)	1.04 (0.99; 1.08)	0.091	0.98 (0.94; 1.03)	0.461
O_3_ (µg/m^3^)	0.93 (0.88; 0.98)	**0.006**	0.92 (0.87; 0.98)	**0.007**
PM10 (µg/m^3^)	1.12 (1.08; 1.17)	**<0.001**	1.08 (1.03; 1.13)	**0.001**
CO (µg/m^3^)	1.02 (0.97; 1.07)	0.518	0.96 (0.91; 1.01)	0.093
1.5 months				
Temperature (°C)	1.02 (1.01; 1.02)	**<0.001**	1.03 (1.02; 1.03)	**<0.001**
Humidity (×10%)	0.98 (0.96; 1.00)	0.084	0.97 (0.96; 0.99)	**0.012**
NO_2_ (µg/m^3^)	1.13 (1.08; 1.19)	**<0.001**	1.14 (1.08; 1.21)	**<0.001**
SO_2_ (µg/m^3^)	1.03 (0.99; 1.07)	0.221	0.98 (0.94; 1.03)	0.451
O_3_ (µg/m^3^)	0.93 (0.88; 0.98)	**0.004**	0.87 (0.83; 0.92)	**<0.001**
PM10 (µg/m^3^)	1.11 (1.06; 1.15)	**<0.001**	1.06 (1.02; 1.11)	**0.003**
CO (µg/m^3^)	1.00 (0.96; 1.05)	0.979	0.96 (0.91; 1.01)	0.110
Two months				
Temperature (°C)	1.02 (1.01; 1.02)	**<0.001**	1.02 (1.01; 1.03)	**<0.001**
Humidity (×10%)	0.98 (0.97; 1.00)	0.126	0.99 (0.97; 1.01)	0.167
NO_2_ (µg/m^3^)	1.09 (1.03; 1.14)	**0.001**	1.13 (1.07; 1.20)	**<0.001**
SO_2_ (µg/m^3^)	1.01 (0.97; 1.05)	0.514	0.99 (0.95; 1.04)	0.808
O_3_ (µg/m^3^)	1.00 (0.96; 1.05)	0.895	0.93 (0.88; 0.98)	**0.006**
PM10 (µg/m^3^)	1.09 (1.05; 1.13)	**<0.001**	1.06 (1.02; 1.10)	**0.005**
CO (µg/m^3^)	0.93 (0.89; 0.97)	**0.001**	0.91 (0.86; 0.96)	**<0.001**

NO_2_, nitrogen dioxide; SO_2_, sulfur dioxide; O_3_, ozone; PM10, particulate matter up to 10 µm in size; CO: carbon monoxide; OR, odds ratio; 95% CI, 95% of confidence interval; PCP, *Pneumocystis* pneumonia. Statistically significant differences are shown in bold.

Table 3 shows the relationship between each environmental factor and death in HIV-positive patients with a PCP diagnosis. In a multivariate CLR analysis, lower temperatures prior to PCP admission, as well as higher concentrations of PM10 at the time of admission were significantly associated with higher likelihoods of dying when two weeks, one month and 1.5 months were used as controls (*p*<0.05) (Table 3). Furthermore, higher concentrations of ozone at one month prior to PCP admission were significantly associated with higher likelihoods of death (*p*=0.011) (Table 3).

**Table 3:** Summary of the association between environmental factors and in-hospital mortality in HIV-positive patients with PCP diagnosis using two weeks, one month, 1.5 months or two months before hospital admission as a control time

	Univariate		Multivariate	
		
Environmental exposure (units)	OR (95% CI)	*p*	OR (95% CI)	*p*
Two weeks				
Temperature (°C)	1.04 (1.00; 1.09)	0.080	1.05 (1.00; 1.10)	**0.047**
Humidity (×10%)	1.02 (0.96; 1.07)	0.583	1.00 (0.95; 1.06)	0.889
NO_2_ (µg/m^3^)	1.16 (1.00; 1.35)	0.057	1.08 (0.9; 1.28)	0.404
SO_2_ (µg/m^3^)	1.03 (0.92; 1.16)	0.565	0.99 (0.88; 1.11)	0.814
O_3_ (µg/m^3^)	0.87 (0.74; 1.03)	0.103	0.92 (0.77; 1.10)	0.373
PM10 (µg/m^3^)	1.22 (1.06; 1.40)	**0.004**	1.16 (1.01; 1.33)	**0.039**
CO (µg/m^3^)	1.17 (1.01; 1.36)	**0.036**	1.11 (0.95; 1.30)	0.199
One month				
Temperature (°C)	1.02 (1.00; 1.05)	0.070	1.04 (1.01; 1.07)	**0.010**
Humidity (×10%)	0.96 (0.91; 1.01)	0.157	0.95 (0.90; 1.00)	0.058
NO_2_ (µg/m^3^)	1.11 (0.96; 1.28)	0.148	1.08 (0.92; 1.27)	0.360
SO_2_ (µg/m^3^)	1.03 (0.92; 1.15)	0.651	0.97 (0.86; 1.09)	0.574
O_3_ (µg/m^3^)	0.83 (0.72; 0.97)	**0.018**	0.80 (0.68; 0.95)	**0.011**
PM10 (µg/m^3^)	1.22 (1.07; 1.39)	**0.002**	1.16 (1.02; 1.33)	**0.030**
CO (µg/m^3^)	1.08 (0.94; 1.23)	0.262	1.03 (0.89; 1.19)	0.713
1.5 months				
Temperature (°C)	1.01 (0.99; 1.03)	0.240	1.02 (1.00; 1.04)	0.048
Humidity (×10%)	0.99 (0.94; 1.04)	0.679	0.98 (0.93; 1.03)	0.455
NO_2_ (µg/m^3^)	1.02 (0.90; 1.16)	0.733	0.99 (0.85; 1.15)	0.869
SO_2_ (µg/m^3^)	1.01 (0.91; 1.12)	0.858	0.96 (0.85; 1.08)	0.450
O_3_ (µg/m^3^)	0.89 (0.78; 1.03)	0.113	0.87 (0.74; 1.01)	0.075
PM10 (µg/m^3^)	1.18 (1.06; 1.33)	**0.004**	1.15 (1.02; 1.30)	**0.021**
CO (µg/m^3^)	1.10 (0.97; 1.24)	0.124	1.08 (0.94; 1.23)	0.291
Two months				
Temperature (°C)	1.01 (0.99; 1.02)	0.490	1.01 (0.99; 1.03)	0.208
Humidity (×10%)	0.99 (0.94; 1.04)	0.591	0.98 (0.93; 1.03)	0.468
NO_2_ (µg/m^3^)	1.01 (0.88; 1.15)	0.911	1.05 (0.90; 1.22)	0.526
SO_2_ (µg/m^3^)	0.98 (0.89; 1.08)	0.716	0.96 (0.86; 1.07)	0.437
O_3_ (µg/m^3^)	0.96 (0.84; 1.09)	0.499	0.91 (0.78; 1.05)	0.190
PM10 (µg/m^3^)	1.11 (0.99; 1.24)	0.074	1.09 (0.97; 1.23)	0.143
CO (µg/m^3^)	0.98 (0.86; 1.11)	0.705	0.95 (0.82; 1.10)	0.515

NO_2_, nitrogen dioxide; SO_2_, sulfur dioxide; O_3_, ozone; PM10, particulate matter up to 10 m in size; CO: carbon monoxide; OR, odds ratio; 95% CI, 95% of confidence interval; PCP, *Pneumocystis* pneumonia. Statistically significant differences are shown in bold.

## Discussion

This manuscript provides a nationwide view of the climatological factors and ambient air pollution that influence PCP epidemiology in HIV-positive patients in Spain. The major findings were: 1) both PCP incidence and mortality dropped by half by the late cART period; 2) a seasonal effect was observed because most hospital admissions and deaths occurred in the colder seasons; and 3) higher concentrations of NO_2_, O_3_, PM10 and CO in the ambient air were significant risk factors for hospital admissions and/or mortality in HIV-positive patients with a PCP diagnosis.

In our study, as expected [4], both the PCP incidence and mortality dropped sharply in the early years of the cART era (1997 to 1999) and then continued to fall throughout the medium (2000 to 2003) and late (2004 to 2011) cART periods. However, PCP remained a major health problem for HIV-positive patients during the last years of the cART era, with still a high number of hospital admissions and deaths. PCP is a common cause of severe disease in HIV-positive patients, especially in patients with low CD4+ T-cells [8,33,34]. cART-derived immune reconstitution is an important protective factor against opportunistic infections [35,36]. In fact, epidemiological surveillance data show that PCP is the second most common AIDS-indicative disease in Spain [37].

Although the MBDS does not provide clinical data on therapy (cART or PCP prophylaxis) nor CD4+ T cell values with which to examine any associations with PCP incidence and mortality, it is possible that patients with PCP were newly diagnosed with HIV at the time or were HIV-positive patients not receiving cART or PCP prophylaxis [1,2,6,34]. Data from the Spanish Ministry of Health show that almost 40% of new HIV diagnoses during 2003 to 2007 had CD4+ values <200 cells/mm^3^ in the first test following diagnosis, and that this characteristic was more common in intravenous drug users (IDU) and heterosexuals [38]. During 2007 to 2011, almost 30% of new HIV diagnoses had CD4+ <200 cells/mm^3^ and 48.1% had CD4+<350 cells/mm^3^ [39]. Also, it must be taken into account that the IDU population has been one of the major drivers of HIV transmission in Spain [40], and this cohort has been associated with limited access to cART, low level of adherence and a high proportion of cART treatment interruption [21,41].

There are other factors related to HIV infection, such as chronic obstructive pulmonary disease (COPD), bacterial pneumonia, pulmonary hypertension, pulmonary fibrosis and *P. jirovecii* colonization of the respiratory tract, that could contribute to increased susceptibility to PCP, and are also potentially affected by climatological factors and ambient air pollution [7]. Additionally, smoking rates are generally high in the HIV-positive population, and long-term exposure to cigarette smoke for HIV-positive individuals may increase the likelihood of complications [42].

The relationship of seasonality and absolute temperature with PCP has been studied for years, however with contradictory findings. Risk of PCP and/or PCP-related mortality has been observed to be higher in winter (Seville, Spain) [15], autumn (London, UK) [16,17], spring (Munich, Germany) [18] and summer (San Francisco, USA) [11]. In our study, we found that incidences of hospital admissions for PCP and PCP-related mortality were both higher during the winter season and during lower absolute temperatures. As suggested by Djawe *et al*. [11], these differences in results could be due to differences in geography, climate, study design, data analysis, patient populations, or *Pneumocystis* genotypes or virulence factors. Because of geographical climate differences, it is possible that winter temperatures in one area are similar to summer temperatures in another area. Spain, the location for our study, is a European country of the Mediterranean basin with several different climates (Mediterranean, oceanic, subtropical and semiarid), but generally the winters are mild, with an average temperature between 10°C and 20°C, and summers are hot, with average temperatures above 30°C in most of the provinces of the country (www.aemet.es/). Thus, the Spanish winter has an average temperature similar to that of other seasons in other countries during which others have found the highest incidences of PCP. Our data, together with previous data, might suggest that rather than a seasonal association, PCP-related outcomes could be highest during the season of the year with average temperatures between 10 and 20 degrees.

To our knowledge, there are only two studies showing data about ambient air pollution and PCP hospital admissions among people living with HIV. In a recent paper, Djawe *et al*. [11] found that increased levels of SO_2_ were associated with PCP hospital admissions in HIV-positive patients. In another paper, Blount *et al*. [13] found that exposure to PM10 and NO_2_ were associated with suppressed IgM responses to *P. jirovecii* in HIV-positive patients that were admitted with PCP, suggesting a mechanism by which ambient air pollution increases host susceptibility to pulmonary infection. In our study, varying concentrations of NO_2_, O_3_, PM10 and CO in the ambient air were significant risk factors for hospital admissions related to PCP. More specifically, higher levels of NO_2_ and PM10 at the time of admission, compared to the control time, were associated with a higher risk of PCP hospital admissions, whereas higher levels of O_3_ and CO at the control time, compared to time of admission, were associated with increased risk of PCP. However, contrary to Djawe *et al*. [11], we did not find any significant data for SO_2_. In relation to respiratory disease hospital admissions, mounting evidence suggests that air pollution contributes to the large global burden of respiratory and allergic diseases including asthma, COPD, pneumonia and possibly tuberculosis [14]. Increased levels of air pollutants have been related to impaired lung function via oxidative stress, which may produce inflammation of the airways and decreased macrophage function and exacerbate existing symptoms [14]. Our findings might suggest that higher levels of air pollution could contribute to an impairment of pulmonary defence mechanisms, thus triggering PCP symptoms, and the need to seek medical care.

As for mortality, we found that only higher ambient levels of O3 and PM10 were related to higher mortality in patients with PCP. In this sense, the climatic factors and air pollution levels showed a weaker association with death than with hospitalization. It is quite likely that environmental factors did not have a direct causal relationship with PCP-related deaths because mortality occurs long after hospital admission. Therefore, the meteorological and air pollution factors might not play a major (or biologically relevant) role in PCP-related mortality, apart from promoting hospital admissions and aggravation of PCP disease.

In our view, this study has a number of advantages that allow the possibility of extrapolating our results to other countries, such as France, Italy and Greece, among others, with similar characteristics as Spain in climate, latitude, contamination levels, prevalence of HIV infection, healthcare coverage, etc. First, this nationwide study was performed in a country with different regional climates throughout the country (Mediterranean, oceanic, subtropical and semiarid) with more than 45 million people living in a large number of cities and small towns. Second, the CCD methodology used to analyze the effects of climate on PCP hospitalizations and deaths has the advantage of not requiring a control group because each patient has two measurements and serves as his or her own control. Thus, some biases related to clinical and demographic characteristics, and geographical variations in Spain were minimized. Third, we used climatological and air pollution data from AEMET (www.aemet.es/), which provides daily average values of the climatological and air pollution variables included in our study for each station throughout the country.

Nevertheless, several aspects must be taken into account for the correct interpretation of the results. First, we had no access to detailed information about the microclimate where each patient was living. Instead of these measurements, we estimated individual exposure levels based on the closest climatological and air pollution monitoring stations. Second, this work was a retrospective study and we had no access to patient clinical data (antiretroviral treatment regimen, duration, adherence, interruptions of cART, CD4+ count, HIV viral load, CDC stage, anti-tuberculosis treatment, etc.), which would be necessary to fully interpret the impact on PCP. Finally, MBDS data are anonymous, and it is not possible to identify whether a patient has been hospitalized at different hospitals within the same calendar year. This may have caused a slight overestimation of our results because we may have considered disease exacerbations or remissions as new participants.

## Conclusions

Our study shows PCP was a significant health problem in the last year of the cART era and its epidemiology was adversely influenced by colder climatological factors (absolute temperature and season) and higher ambient air pollution (NO_2_, O_3_, PM10 and CO levels).
